# Outcomes in skilled nursing facilities versus other locations in outpatient parenteral antimicrobial therapy among patients with substance use disorders

**DOI:** 10.1017/ash.2026.10364

**Published:** 2026-04-27

**Authors:** Armani M. Hawes, Lisa R. Yanek, Megan E. Buresh, Rawan Abdel-Galil, Alia Bodnar, Oluwaseun O. Falade-Nwulia, Sara Condron Keller

**Affiliations:** 1 Department of Medicine, Division of Infectious Diseases, Johns Hopkins University School of Medicine, Baltimore, MD, USA; 2 Department of Medicine, Division of General Internal Medicine, Johns Hopkins University School of Medicine, Baltimore, MD, USA; 3 Department of Medicine, Division of Addiction Medicine, Johns Hopkins University School of Medicine, Baltimore, MD, USA

## Abstract

**Objective:**

People with substance use disorder (SUD) make up an increasing proportion of patients hospitalized for infections that require outpatient parenteral antimicrobial therapy (OPAT). In many settings, patients with SUD may be unable to receive home-based OPAT, and so stay in the hospital or get discharged to skilled nursing facilities (SNF) for ongoing treatment. Our objective was to compare outcomes, especially engagement in SUD treatment, among patients with SUD who received OPAT in SNF versus other settings.

**Design:**

Retrospective cohort study.

**Setting:**

Two academic medical centers.

**Patients:**

Patients with a history of SUD and discharged on OPAT.

**Methods:**

We used a multivariate logistic regression to determine predictors of outcomes among patients discharged to SNF versus other locations.

**Results:**

Among 350 patients with SUD discharged on OPAT, 285 (81.4%) were discharged to SNF. Hospital readmissions, emergency department visits, infection relapse, catheter complications, and adverse drug events related to OPAT were similar in the two groups. Median length of stay was longer in patients discharged to SNF (16 d vs 12 d, P = .001). Being discharged to a SNF was associated with a lower likelihood of engaging in SUD treatment at 30 days postdischarge (adjusted odds ratio: 0.48, 95% confidence interval: 0.26–0.87).

**Conclusions:**

Patients with SUD requiring OPAT discharged to SNF may have decreased engagement in SUD treatment.

## Introduction

People with substance use disorders (SUD), particularly those with opioid use disorder (OUD), are at high risk of hospitalization for infections.^1,2^ For example, hospitalizations with injection-related endocarditis increased twelve-fold from 2010 to 2015, and as many as 20% of patients with injection opioid use may die of endocarditis.^3–5^ Injection-related infections may include conditions such as bacteremia, septic arthritis, osteomyelitis, endocarditis, and skin and soft tissue infections.^6,7^ A hospitalization for a serious infection can be an opportunity to engage or reengage in SUD care. However, many patients with SUD do not receive SUD treatment during their hospitalization, leading to missed opportunities for SUD care engagement, readmissions, and increased healthcare costs.^8–11^ Many injection-related infections require long-term parenteral antimicrobial therapy, often provided postdischarge as outpatient parenteral antimicrobial therapy (OPAT). Patients with SUD receiving OPAT versus patients without SUD tend to have longer lengths of stay, more patient-directed discharges (PDD), and increased readmissions.^12^

Most patients on OPAT receive parenteral therapy in their home with assistance from home infusion therapy.^13^ Patients on OPAT often prefer receiving treatment at home, where they can be in more comfortable and familiar surroundings, when compared to skilled nursing facilities (SNF).^14^ While studies of patients with SUD suggest that many can be safely treated in the home with central venous catheters (CVCs) with appropriate addiction treatment,^15,16^ many home infusion agencies have policies that preclude patients with SUD from being treated in their homes, due to concerns for misuse of the CVC.^17^ Meanwhile, patients receiving OPAT in SNFs are more likely to experience process failures such as non-receipt of laboratory monitoring and poorer follow-up than those receiving OPAT at home, possibly related to communication barriers, and potentially as a result, are more likely to be readmitted within 30 days of hospital discharge.^13,14,18,19^ Understanding outcomes among patients with SUD requiring OPAT discharged to SNF is important in making decisions about appropriate locations to receive OPAT (eg, home, SNF, residential treatment facility, infusion clinic, etc.), as well as whether other options for infectious disease management may be appropriate (eg, long-acting injectable antimicrobials, oral antimicrobials, etc.). In this work, we aimed to compare engagement in SUD treatment at 30 days and other outcomes among patients with SUD discharged on OPAT to SNF compared to other locations.

## Methods

In this retrospective cohort study, we reviewed records from patients with SUD who were discharged on OPAT from two academic medical centers in Baltimore, Maryland from April 2020 through April 2024. The Johns Hopkins University School of Medicine Institutional Review Board declared the work quality improvement/non-human subjects research (IRB00389870).

The OPAT Program encompasses both hospitals, and includes infectious diseases specialty physicians, pharmacists, and nurses, as well as pharmacy technicians and medical assistants. The OPAT Program manages patients discharged on OPAT to home, SNF, hemodialysis, prison or jail, infusion clinics, or other settings. A subset of patients with SUD, including those with a recent history of injecting drugs, can receive OPAT in a home setting via one home infusion company if there was insurance coverage, the patient would otherwise qualify for home infusion, the patient has a safe home setting, the patient is engaged in SUD care, and the patient is agreeable to home-based OPAT.

Both hospitals also have addiction consult services (ACS) comprised of addiction medicine providers, pharmacists, social workers and peer recovery specialists (PRS). PRS are individuals with lived experience of substance use and recovery who provide recovery support and connect patients to SUD treatment and other community-based resources patients can access posthospitalization. ACS clinicians provide recommendations on medication for opioid use disorder (MOUD) initiation or dose titration, as well as other medications for addiction treatment.

Patients included in this study were those who had received an infectious diseases consultation and diagnosed with an infection requiring OPAT, who also had a SUD diagnosis on their electronic problem list (eg, alcohol use disorder, OUD, stimulant use disorder, cocaine use disorder, tobacco use disorder, cannabis use disorder, or other illicit SUD; in remission or ongoing) in the electronic health record (EHR) and confirmed upon chart review. Patients discharged on long-acting injectable antimicrobial agents, patients discharged on complex oral outpatient antimicrobial therapy (COpAT), patients with PDD from the hospital prior to arrangement of OPAT, and children under the age of 18 were excluded.

The primary exposure was receipt of OPAT in a SNF. Demographic and clinical information regarding the patient’s infection and treatment including indication, SUD history and treatment, and discharge location to SNF or to other settings (eg, to home with or without additional support for SUD, to hemodialysis, to prison or jail, etc.) were abstracted. We further stratified SUD as ongoing (ie, within the last six months) or in remission. SUD treatment engagement could include medication-assisted or non-medication-assisted treatment in the community (eg, mutual aid organizations, inpatient treatment, outpatient treatment, residential treatment, intensive outpatient treatment, etc.). Charts were abstracted by three physicians (A.H., S.K., M.B.) and a trained research assistant (R.A.G.); a random sample of 20% of charts were re-reviewed by a physician.

Outcomes included all-cause readmissions within 30 days of discharge or between 31 and 90 days of discharge, ED visits within 30 days of discharge, length of hospital stay, receipt of required laboratory tests by the OPAT team, infection relapse within 90 days,^20^ catheter complications,^20^ OPAT-related adverse drug events,^20^ and engagement in SUD treatment at 30 days. We also reported PDD from SNF as noted in the EHR and as recorded by the OPAT Program (who track down labs and manage appointments, and who attempt to call each SNF at least weekly). For engagement in SUD treatment at 30 days, if no documentation of community SUD treatment (including medication-assisted or non medication-assisted treatment) was in the chart, the patient was considered to not be engaged in treatment.

Descriptive statistics were used to assess characteristics of those discharged to SNF compared to those not discharged to SNF. Comparisons between groups of interest were tested using χ^2^ or Fisher’s exact tests for categorical variables and *t*-tests or Wilcoxon rank-sum tests for continuous variables. We also performed multivariate analyses examining the predictors of being engaged in SUD treatment 30 days postdischarge, focusing on patients discharged to SNF versus to other locations. Variables were considered for inclusion in the multivariate analysis via backward elimination. All analyses were conducted in Stata Ver. 15 (Stata Inc, College Station, TX).

## Results

We reviewed records from 350 patients with SUD who were discharged on OPAT. Of these, 285 (81.4%) were discharged to a SNF while 65 (18.6%) were discharged to other locations including 4 (1.1%) discharged to a substance use residential treatment facility, 38 (10.9%) to a home-based OPAT program designed for patients with SUD, 9 (2.6%) to home without a specific program for patients with SUD, 13 (3.7%) to hemodialysis, and 1 (0.3%) to prison (Table 1). Fewer patients discharged to SNF had HD access (1.8% vs 18.5%, *P* < .001). More patients discharged to SNF were discharged on vancomycin (46.3% vs 30.9%, *P* = .049), while fewer patients discharged to SNF were discharged on a parenteral antifungal (0.3% vs 4.6%, *P* = .004). More patients discharged to SNF had reported any prior injection drug use (IDU, 79.3% vs 66.2%, *P* = .023) and IDU within the prior six months (66.3% vs 29.2%, *P* < .001). More patients discharged to SNF used opioids (90.5% vs 70.8%, *P* < .001), cocaine (59.7% vs 26.2%, *P* < .001) and tobacco (50.9% vs 30.8%, *P* = .003). More patients discharged to SNF had been seen by the ACS (79.7% vs 53.8%, *P* < .001) or PRS (55.1% vs 30.8%, *P* = <.001).

**Table 1.** Descriptions of patients living with substance use disorders requiring OPAT receiving OPAT at SNF vs other location

Characteristic	Discharged to SNF, N = 285 (%)	Discharged to other location* N = 65 (%)	Total, N = 350 (%)	*P*-value for difference
Female	118 (41.6)	28 (43.1)	146 (41.8)	.82
Race: White non-Hispanic	194 (68.1)	39 (60.0)	233 (66.6)	.31
Black non-Hispanic	78 (27.4)	24 (36.9)	102 (29.1)	–
Hispanic or other	14 (4.9)	3 (4.6)	17 (4.86)	–
Age, mean (standard deviation) years	45.8 (11.9)	47.4 (12.4)	46.1 (12.0)	.30
Insurance private (uninsured = 1)	32 (11.2)	12 (18.5)	44 (12.6)	.11
Medicaid	211 (74.0)	40 (61.5)	251 (71.7)	.80
Medicare	41 (14.4)	13 (20.0)	54 (15.4)	.26
Type of access: HD access	5 (1.8)	12 (18.5)	17 (4.9)	<.001
Tunneled CVC	50 (17.5)	12 (18.5)	62 (17.7)	–
Midline	7 (2.5)	2 (3.1)	9 (2.6)	–
PICC	223 (60.0)	39 (78.3)	262 (74.9)	–
Type of antibiotic: penicillins	71 (24.9)	18 (27.7)	89 (25.4)	.64
Cephalosporin	118 (41.4)	26 (40.0)	144 (41.1)	.84
Carbapenem	11 (3.9)	2 (3.1)	13 (3.7)	.76
Vancomycin	132 (46.3)	21 (30.9)	153 (43.7)	.049
Daptomycin	11 (3.9)	2 (3.1)	13 (3.7)	.76
Antifungal (micafungin = 3, liposomal amphotericin = 1 non-SNF patient)	1 (0.3)	3 (4.6)	4 (1.1)	.0035
Indication for OPAT: bacteremia	65 (22.8)	15 (23.1)	80 (22.9)	.96
Endocarditis	43 (15.1)	9 (13.9)	52 (14.9)	.80
Endovascular infection	13 (4.6)	0 (0.0)	13 (3.7)	.079
Meningitis	5 (1.8)	5 (7.7)	10 (2.9)	.010
Osteomyelitis	139 (48.8)	26 (40.0)	165 (47.1)	.20
Discitis	32 (11.3)	2 (3.1)	34 (9.7)	.045
Epidural abscess	12 (4.2)	0 (0)	12 (3.4)	.092
Septic arthritis	51 (17.9)	5 (7.7)	56 (16.0)	.043
Orthopedic hardware infection	40 (14.0)	11 (16.9)	51 (14.6)	.55
Pneumonia or empyema	7 (2.5)	1 (1.5)	8 (1.3)	.66
Intraabdominal abscess	5 (1.8)	3 (4.6)	8 (2.3)	.16
Skin and soft tissue infection	11 (3.9)	5 (7.7)	16 (4.6)	.18
Addiction medicine consultation	227 (79.7)	35 (53.8)	262 (74.9)	.0001
Peer recovery support consultation	157 (55.1)	20 (30.8)	177 (50.6)	.0004
Type of substance use (other = 28): opioid	258 (90.5)	46 (70.8)	304 (86.9)	<.001
Benzodiazepine	21 (7.4)	2 (3.1)	23 (6.6)	.21
Alcohol	51 (17.9)	15 (23.1)	66 (18.9)	.34
Cocaine	170 (59.7)	17 (26.2)	187 (53.4)	<.0001
Cannabis	53 (18.6)	7 (10.8)	60 (17.4)	.13
Tobacco (8 used tobacco as only substance)	145 (50.9)	20 (30.8)	165 (47.1)	.003
Any injection drug use	226 (79.3)	43 (66.2)	269 (76.9)	.0234
Injection drug use in past 6 months	189 (66.3)	19 (29.2)	208 (59.4)	<.001
Prescription for MOUD at hospital discharge, among patients with OUD (not applicable N = 46)	230 (89.2)	37 (80.4)	267/304 (87.8)	.096
Buprenorphine	66 (25.6)	8 (17.4)	74 (24.3)	.23
Methadone	163 (63.2)	29 (63.0)	192 (63.2)	.99
Length of hospitalization (median, interquartile range) days	16 (10, 23)	12 (7, 18)	15 (9, 22)	<.001

* Substance use residential treatment facility: N = 4, 1.1%; Home with OPAT program for patients with SUD: N = 38, 10.9%; Home without OPAT program for patients with SUD: N = 9, 2.6%; Prison: N = 1, 0.3%; Hemodialysis facility: N = 13, 3.7%.

Outcomes were similar between the two groups (Table 2). There were no statistically significant differences in hospital readmission within 30 days (19.7% vs 21.5%, *P* = .73), hospital readmission between 31 and 90 days (32.0% vs 23.1%, *P* = .18), emergency department visits within 30 days (14.7% vs 18.5%, *P* = .45), infection relapse within 90 days (5.6% vs 9.2%, *P* = .28), catheter complications (8.1% vs 12.3%, *P* = .28), or adverse drug events related to OPAT (6.0% vs 4.6%, *P* = .67). However, median length of hospital stay was longer in the group discharged to SNF (16 d vs 12 d, *P* = .001). Almost one in four (24.1%) patients discharged to SNF experienced a PDD.

**Table 2.** Outcomes among patients requiring OPAT with SUD discharged to SNF or to other locations

Characteristic	Discharged to SNF, N = 285 (%)	Discharged to other location, N = 65 (%)	Total, N = 350 (%)	*P*-value for difference
First set of labs received by OPAT team	170 (60.7)	34 (52.3)	204 (59.1)	.31
Hospital readmission within 30 days	56 (19.7)	14 (21.5)	70 (20.1)	.73
Hospital readmission between 31 and 90 days	91 (32.0)	15 (23.1)	106 (30.4)	.18
Emergency department visit within 30 days of discharge	42 (14.7)	12 (18.5)	54 (15.4)	.45
Infection relapse within 90 days	16 (5.6)	6 (9.2)	22 (6.3)	.28
Catheter complication*	23 (8.1)	8 (12.3)	31 (8.9)	.28
OPAT-related adverse drug events	17 (6.0)	3 (4.6)	20 (5.7)	.67
Engaged in substance use disorder treatment at 30 days	138 (48.4)	40 (61.5)	178 (50.9)	.058
Patient-directed discharge from skilled nursing facility	66 (24.1)	N/A	N/A	N/A

* Catheter complications include catheter fracture (N = 1), exit site infection (N = 1), occlusion (N = 3), venous thromboembolism (N = 1), catheter-related bloodstream infection (N = 5), bloodstream infection (N = 4), inadvertent removal (N = 5), catheter tampering (N = 12).

We also investigated likelihood of engaging in SUD treatment at 30 days postdischarge (Table 3). We found that when adjusted for OUD, tobacco use disorder, and IDU within the prior six months, being discharged to a SNF was associated with a lower likelihood of engaging in SUD treatment at 30 days postdischarge (adjusted odds ratio: 0.48, 95% confidence interval: 0.26–0.87).

**Table 3.** Associations with engaging in substance use disorder treatment at 30 days

Variable	Odds ratio of engaging in substance use disorder treatment at 30 days (95% confidence interval)	Adjusted odds ratio of engaging in substance use disorder treatment at 30 days (95% confidence interval)*
Discharge to SNF	0.59 (0.34–1.02)	0.48 (0.26–0.87)
Insurance: Medicaid (vs private)	0.91 (0.41–2.03)	N/A
Medicare (vs private)	0.94 (0.49–1.78)	N/A
Gender: Female (vs male)	0.72 (0.47–1.10)	N/A
Race or ethnicity: Black vs white non-Hispanic	0.52 (0.19–1.46)	N/A
Hispanic or other race vs white non-Hispanic	1.08 (0.68–1.72)	N/A
Vancomycin prescription	1.06 (0.69–1.61)	N/A
Opioid use disorder	1.56 (0.83–2.92)	1.76 (0.85–3.63)
Cocaine use disorder	1.09 (0.72–1.66)	N/A
Tobacco use disorder	1.10 (0.72–1.67)	1.19 (0.77–1.84)
Benzodiazepine use disorder	1.06 (0.45–2.47)	N/A
Cannabis use disorder	0.96 (0.55–1.67)	N/A
Injection drug use within prior 6 months	1.16 (0.76–1.79)	1.14 (0.70–1.88)
Any injection drug use	1.49 (0.90–2.46)	N/A

* Multivariate model included discharge to SNF, opioid use disorder, tobacco use disorder, and injection drug use within prior 6 months.

## Discussion

We found that among patients with SUD requiring OPAT, there were no statistically significant differences in hospital readmission, emergency department visits, infection relapse, or complications for patients discharged to SNF compared to patients discharged elsewhere. However, length of hospital stay was prolonged in the group going to SNF, and patients discharged to SNF were less likely to be engaged in SUD treatment 30 days postdischarge.

Prior work has also suggested that patients with SUD requiring OPAT who were discharged to SNF experience high rates of healthcare services use, including 30-day hospital readmissions as high as 29%.^21^ Our work adds to this by being one of the first to directly compare patients with SUD requiring OPAT discharged to SNF versus other locations (primarily home), and further suggest that patients with SUD may have similar outcomes whether they are discharged to SNF or not. One other study similarly failed to show a difference in outcomes in patients discharged to SNF on OPAT,^12^ but that study only followed 52 patients.

Other data suggest that patients discharged to SNF on OPAT have higher rates of non-receipt of laboratory monitoring and poorer follow-up than those receiving OPAT at home.^18,19^ In our OPAT Program, we focus on obtaining laboratory test results from all locations patients are discharged to, which may have made us more successful in receiving laboratory test results from SNF. A strong OPAT program may mitigate some struggles noted with laboratory test result receipt in prior studies.^13^ However, in our workflows, receiving laboratory test results from SNFs is extremely time-consuming.

In this study, patients with SUD requiring OPAT discharged to SNF versus to other locations had longer lengths of hospital stay. Some of this is likely due to the additional logistical work needed to identify an accepting SNF, although the extended length of hospital stay may also partially reflect infection severity and medical complexity. Previous work has shown that patients with SUD have lower rates of SNF acceptance and a higher likelihood of being accepted to lower quality facilities.^17,22,23^ Taken together, these data suggest that patients with SUD requiring OPAT may have similar infection-related outcomes and shorter lengths of stay if they are discharged to a location other than a SNF if they otherwise qualify for a location other than a SNF.

In addition, we noted that patients discharged to SNF were less likely to still be receiving SUD treatment at 30 days posthospital discharge. This is not surprising, as there are multiple barriers that preclude arranging long-term SUD treatment until completion of the SNF stay, leading to delayed entry to SUD treatment. Patients discharged to SNF may have also had fewer social or economic resources to support ongoing engagement in SUD treatment. In our experience, most SNFs do not provide any SUD support or treatment.^22^ Mean length of stay at SNFs in one study was 21 days;^24^ it is possible that some patients in our study may have then engaged in SUD treatment after discharge from SNF, but most SNFs also do not provide coordination services to ensure SUD care after discharge from SNF. At our hospitals, hospital-based PRS do not have capacity to support patients after hospital discharge, and cannot make direct referrals to community-based SUD treatment for patients being discharged to SNF, other than for outpatient treatment programs for continuation of methadone. In our clinical experience, the SNF stay often is a time when SUD treatment is lacking, with no treatment besides continuation of medication started in the hospital (ie, unlikely to have dose adjustments of MOUD), and no groups or other supports as well as risks of gaps in MOUD. A previous pilot included grant funding for a SNF-embedded PRS showed promise for increased uptake of MOUD during and after SNF stay, but more sustainable solutions are required.^25^ We are exploring opportunities to fill this gap and better support continued SUD treatment by including outpatient PRS in care prior to discharge, and this remains an important area of study.

We also noted that one in four patients with SUD on OPAT discharged to SNF experienced a PDD. Other studies have shown that patients with SUD requiring OPAT frequently experienced PDD from SNF (20%) or otherwise left a SNF prior to the scheduled completion of therapy (16%).^26^ Further investigations should explore whether and why patients with SUD requiring OPAT frequently experience PDD from SNF. Of note, we may have underestimated PDD in this analysis, as we did not have access to all of the SNF records and instead relied on OPAT Program members documenting that patients had experienced PDD when reaching out to SNFs for care coordination purposes.

Our study had limitations. We performed a retrospective review of medical records at two medical centers in one health care system with an ACS and an OPAT program, and some of the patients who received OPAT at home were enrolled in a program with a focus on SUD. It is possible that these results may not be easily translated to other health care systems. Similarly, our study only included patients who required OPAT, not including patients on complex outpatient antimicrobial therapy (COpAT) or long-acting injectable antimicrobials. However, for many patients with SUD requiring OPAT, alternatives to OPAT (such as COpAT, or long-acting injectable antimicrobial) may be more appropriate. We also may have underestimated SUD treatment engagement, as well as PDD, although the OPAT Program had ongoing communication with SNFs that allowed some of this data to be captured. The study population also did not include patients who experienced PDD from the hospital prior to initiating OPAT. There were fewer patients in the non-SNF group which may have limited the power to detect differences. We lacked data on housing status and other socioeconomic factors such as access to transportation that might influence postdischarge treatment options and the ability to engage in ongoing SUD or OPAT treatment. We did not include data on ACS in the final multivariate analysis of engagement in care as we felt that this was on the pathway of treatment rather than a confounder; however, it is important to note that more patients going to SNF had an inpatient addiction consultation. We used approaches to confirm engagement in care, but may not have fully counted all patients engaged in care. Finally, there is likely a difference between patients who may require SNF versus those who may not require SNF (eg, medical complexity, severity of infection, increased needs, unstable housing or housing status), and there is likely a subset of the patients attending SNF who would not have been eligible for OPAT at other locations even in the absence of SUD. This may have led to selection bias.

Our data adds to the growing literature reflecting a lack of increased risk of poor outcomes for selected patients with SUD who may be able to receive OPAT in other settings (eg, the home, infusion centers, addiction treatment centers, or hemodialysis), and patients receiving OPAT in other settings may have decreased lengths of hospital stay. Some barriers may exist; for example, lack of housing may preclude home OPAT as a treatment option. Ongoing partnerships with SUD treatment facilities may also present an option for OPAT delivery. In addition, as patients may be more likely to be engaged in SUD treatment if they do not go to a SNF, and as a significant proportion of patients with SUD discharged to SNF on OPAT in our study experienced PDD, there may be occasions when patients with SUD requiring OPAT would benefit from not going to a SNF. We suggest that decisions about the best treatment approach should be patient centered and multidisciplinary, accounting for the risks and benefits of each treatment location for the individual patient. More health systems-based work, including embedding OPAT into residential addiction treatment facilities and expanding access to home-based OPAT for patients with SUD, is needed to expand access to patient-centered options for completion of required antimicrobial therapy for patients with SUD.

## Data Availability

Data available upon request.

## Administrative, technical, or material support

Biostatistics, Epidemiology, and Data Management (BEAD) Core, Johns Hopkins School of Medicine, Baltimore, MD.
